# Toll like receptor (TLR)-4 as a regulator of peripheral endogenous opioid-mediated analgesia in inflammation

**DOI:** 10.1186/1744-8069-10-10

**Published:** 2014-02-06

**Authors:** Reine-Solange Sauer, Dagmar Hackel, Laura Morschel, Henrike Sahlbach, Ying Wang, Shaaban A Mousa, Norbert Roewer, Alexander Brack, Heike L Rittner

**Affiliations:** 1 Department of Anesthesiology, University Hospital of Wuerzburg, Oberdürrbacher Strasse 6, D-97080 Würzburg, Germany; 2 Department of Anesthesiology and Operative Intensive Care, Campus Benjamin Franklin, Charité, Universitätsmedizin Berlin, Hindenburgdamm 30, D-12200 Berlin, Germany; 3 Department of Anesthesiology and Operative Intensive Care, Charité, Universitätsmedizin Berlin, Augustenburger Platz 1, D-13535 Berlin, Germany

**Keywords:** Toll like receptors, Analgesia, Inflammatory pain, Endogenous opioids

## Abstract

**Background:**

Leukocytes containing opioid peptides locally control inflammatory pain. In the early phase of complete Freund’s adjuvant (CFA)-induced hind paw inflammation, formyl peptides (derived e.g. from *Mycobacterium butyricum*) trigger the release of opioid peptides from neutrophils contributing to tonic basal antinociception. In the later phase we hypothesized that toll-like-receptor-(TLR)-4 activation of monocytes/macrophages triggers opioid peptide release and thereby stimulates peripheral opioid-dependent antinociception.

**Results:**

In Wistar rats with CFA hind paw inflammation in the later inflammatory phase (48–96 h) systemic leukocyte depletion by cyclophosphamide (CTX) or locally injected naloxone (NLX) further decreased mechanical and thermal nociceptive thresholds. *In vitro* β-endorphin (β-END) content increased during human monocyte differentiation as well as in anti-inflammatory CD14^+^CD16^-^ or non-classical M2 macrophages. Monocytes expressing TLR4 dose-dependently released β-END after stimulation with lipopolysaccharide (LPS) dependent on intracellular calcium. Despite TLR4 expression proinflammatory M1 and anti-inflammatory M2 macrophages only secreted opioid peptides in response to ionomycin, a calcium ionophore. Intraplantar injection of LPS as a TLR4 agonist into the inflamed paw elicited an immediate opioid- and dose-dependent antinociception, which was blocked by TAK*-*242, a small-molecule inhibitor of TLR4, or by peripheral applied NLX. In the later phase LPS lowered mechanical and thermal nociceptive thresholds. Furthermore, local peripheral TLR4 blockade worsened thermal and mechanical nociceptive pain thresholds in CFA inflammation.

**Conclusion:**

Endogenous opioids from monocytes/macrophages mediate endogenous antinociception in the late phase of inflammation. Peripheral TLR4 stimulation acts as a transient counter-regulatory mechanism for inflammatory pain *in vivo*, and increases the release of opioid peptides from monocytes *in vitro*. TLR4 antagonists as new treatments for sepsis and neuropathic pain might unexpectedly transiently enhance pain by impairing peripheral opioid analgesia.

## Background

Inflammatory pain is elicited by proalgesic mediators e.g. proinflammatory cytokines, prostaglandins, and bradykinin [1,2]. Recently, toll like receptors (TLR) have been identified as important mediators in inflammatory as well as neuropathic pain [3,4]. TLRs are pattern recognition receptors (PAR) recognizing exogenous foreign material like bacteria or viruses. Direct stimulation of TLR with exogenous ligands e.g. lipopolysaccharide (LPS) - or possibly mycobacteria binding TLR4 - can provoke pain [5-7]. Pain is decreased in TLR2 or −4 deficient mice with neuropathic lesions [8,9], which implies TLR activation via endogenous ligands like heat shock proteins. Most of the proalgesic action of TLRs is due to sensory neurons detecting and relaying pain messages in response to local peripheral inflammation, mostly attributed to the spinal level including activation of microglia and sensory neurons [3]. Peripheral mechanisms are less studied so far. Since TLR4 antagonists are proposed to enter clinical trails for treatment sepsis as well as neuropathic pain a careful evaluation of these is important for the design of the studies [10].

Intraplantar injection of heat inactivated *Mycobacterium butyricum* in oily solution (“complete Freund’s adjuvant, CFA”) elicits inflammatory pain resulting in spontaneous activity of nociceptive Aδ and C nerve fibers [1,11]. The net intensity of inflammatory pain is not only dependent on proalgesic mediators, but is counterbalanced by endogenous analgesic mediators including opioid peptides [12]. In early CFA inflammation neutrophils predominate. Stimulation of neutrophils by chemokines like CXCL2/3 or bacterial components (formyl peptides) induces release of opioid peptides like β-endorphin (β-END) and met-enkephalin (Met-ENK). This counterbalances hyperalgesic mediators and induces peripheral opioid-mediated antinociception in models of neuropathic, inflammatory, arthritic, bone cancer and visceral pain [13-17]. So far T-lymphocytes and neutrophils have been studied in detail [18-20]. Antigen-specific and stimulated T-cells increase the synthesis of met-enkephalin and elicit antinociception in adoptive transfer modes in arthritic pain. Mycobacteria stimulate neutrophils to secret of opioid peptides via formyl peptide receptors, although we expected stimulation via TLR2 and TLR4, because these are the primary target receptors of mycobacteria [21]. However, monocytes are not stimulated to release opioid peptides by mycobacteria *in vitro* although they express formyl peptide receptors*.* Monocytes/macrophages are the major opioid containing leukocyte population after 48 h CFA inflammation [22]. Moderate depletion of monocytes/macrophages by clodronate liposomes reduces swim stress-induced peripheral opioid-mediated analgesia [23]. To sum up, the role of opioid-containing monocytes/macrophages has not been clearly defined in inflammatory pain.

Monocytes are divided in classical pro-inflammatory CD14^+^CD16^+^ and anti-inflammatory CD14^+^CD16^-^ monocytes. CD14^+^CD16^+^, in contrast to CD14^+^ non-expressing CD16 classical monocytes, represent <10% of the total monocytes in normal blood but increase in various inflammatory diseases [24]. Monocytes migrate into the tissue and differentiate into macrophages. *In vitro* macrophages can be functionally polarized by granulocyte macrophage colony-stimulating factor (GM-CSF) into M1 (proinflammatory, antimicrobial) and by macrophage colony-stimulating factor (M-CSF) and interleukin-10 (IL-10) into M2 (alternatively activated) for tissue repair. TLR agonists and lipopolysaccharide (LPS) are important activators of monocytes and macrophages. In addition macrophages express a variety of other surface markers in part of unknown function including PM-2 K and 25 F9 [25].

The present study examines molecular mechanisms of peripheral opioid-mediated analgesia in late CFA inflammation. Specifically, we investigated i) the subtypes of monocytes and macrophages for *in vitro* opioid peptide release after TLR4 stimulation and ii) the characteristics of LPS-induced antinociception and iii) involvement of TLR4 in the generation of tonic peripheral antinociception.

## Results

### Immune cells and peripheral opioids contribute to baseline analgesia in inflammatory pain

**Figure 1:**
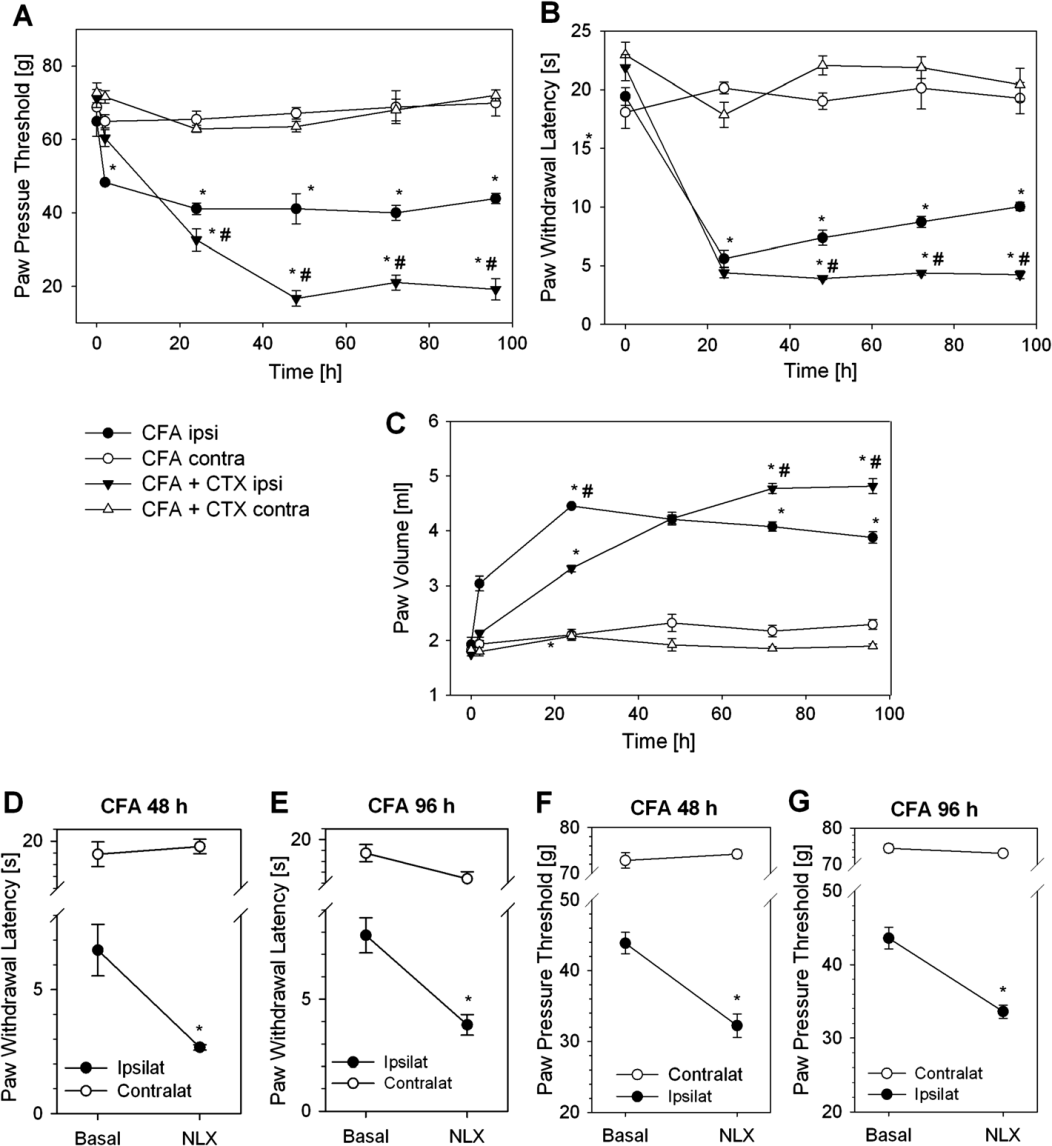
**Increased hyperalgesia by leukocyte depletion or peripheral opioid receptor blockade in inflammatory pain.** Male Wistar rats were leukocyte depleted with cyclophosphamide (CTX, 100 mg/kg and 50 mg/kg BW on day −3, -1 and 1). Complete Freund’s adjuvant (CFA) was injected i.pl. at day 0. Paw pressure thresholds **(A)**, paw withdrawal latency **(B)** and paw volume **(C)** were measured at 0, 24, 48, 72 and 96 h after induction of inflammation in the ipsilateral (ipsi) and contralateral (contra) paw (no CTX n = 3–6, CTX n = 6–8,. * p < 0.05 vs. 0 h, # p < 0.05 vs. CFA ipsilateral, Two Way RM ANOVA, Student Newman Keuls). CTX alone did not change paw pressure thresholds [28]. **(D, F)** After 48 h and **(E, G)** 96 h of CFA hind-paw inflammation the opioid receptor antagonist naloxone (NLX, 0.56 ng) was injected i.pl. Thermal **(D, E)** and mechanical **(F, G)** nociceptive thresholds were determined before and 10 min after treatment and compared to contralateral side (n = 4–6, * p < 0.05, * vs. basal, Two Way ANOVA, Student-Newman-Keuls). No change was seen in saline injected rats before [14]. All data are presented as MEAN ± SEM.

Treatment with cyclophosphamide (CTX) significantly depletes immune cells in the blood in Wistar rats [26]. In rats with 96 h CFA-induced hind-paw inflammation immune cell depletion with CTX reduced RP1^+^CD45^+^-neutrophils by 96.6% (340.0 ± 84.2 vs. 11.5 ± 1.9 × 10^3^ cells), ED1^+^CD45^+^-macrophages by 97.3% (806.8 ± 145.1 vs. 22.0 ± 3.0 × 10^3^ cells) and 3E7^+^CD45^+^-opioid containing leukocytes by 93.1% (455.8 ± 103.0 vs. 31.4 ± 5.4 × 10^3^ cells) in the inflamed paw as previously measured by flow cytometry [27]. CTX treatment further lowered mechanical (Figure 1A) and thermal (Figure 1B) nociceptive thresholds in the inflamed paw beginning on day one after intraplantar injection of CFA while the contralateral paws were unaffected. However, the kinetics of the effect was different: Mechanical nociceptive thresholds steadily decreased up to 48 h and then remained stable in CTX treated rats. Thermal nociceptive thresholds were similar between CFA ± CTX as well as CFA only rats at 24 h, but in CFA only rats paw withdrawal latency progressively rose over time, while CFA ±  CTX remained low. No change in paw pressure thresholds or paw withdrawal latencies was previously seen in rats treated CTX alone [28]. Paw volume was significantly less in early inflammation (24 h post CFA) in immunodepleted rats, but paws were significantly more swollen in late inflammation (72–96 h post CFA) in rats with CTX treatment (Figure 1C). No change of paw volume was observed in the contralateral paw.

Intraplantar injection of naloxone (NLX) in a low dose can evaluate the contribution of peripheral opioid receptors because central receptors are not blocked [14,29]. Injection of NLX (1.8 μg) 48 and 96 h after induction of inflammation further significantly reduced thermal (Figure 1D, E) and mechanical (Figure 1F, G) nociceptive thresholds. No change in paw withdrawal latency or paw pressure threshold was seen in the contralateral paw (Figure 1D-G) as well as by injection of solvent (saline) only in previous paw withdrawal latency experiments [14] as well as current paw pressure threshold measurements (CFA 48 h saline injection: basal 43.6±1.2 g; treated 43.0±1.1 g; CFA 96 h saline injection: basal 42.7±1.3 g; treated 44.2±1.1 g). During CFA inflammation neutrophils are the predominant source of opioid peptides in early stages of inflammation (2–6 h) while monocytes/macrophages are the major source at later stages (96 h) [22,23]. Therefore, we hypothesized that monocytes and macrophages could be the source of peripheral opioid mediated analgesia.

### Macrophage subpopulation in the inflamed paw

**Figure 2:**
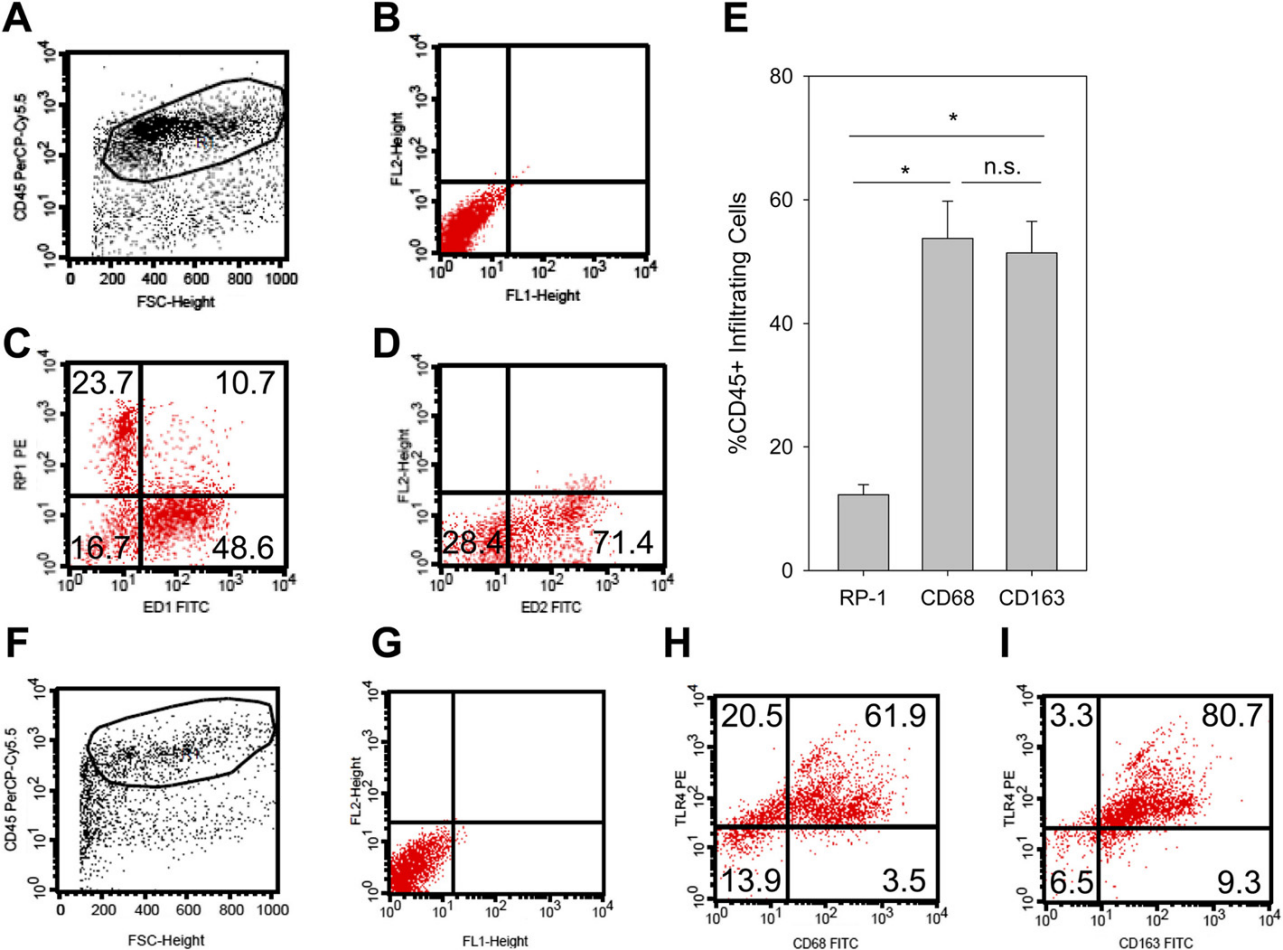
**Characterization of infiltrating leukocytes in the inflamed paw. Rats were injected with CFA for 4 d. (A)** Cells suspensions from subcutaneous paw tissue were first stained for CD45 to gate on hematopoetic cells (x-axis: forward scatter [size], y-axis: CD45-PerCp-Cy5). **(B)** The dot plot (x-axis: FITC, y-axis: PE) shows unstained controls from cells gated on CD45. CD45^+^ cells were stained for RP-1 (neutrophils), CD68 (M1, proinflammatory macrophages) (**C,** x-axis: CD68-FITC, y-axis: RP-1-PE) as well as and CD163 (M2, antiinflammatory macrophages) (**D,** x-axis: CD163-FITC, y-axis: PE). Representative examples are shown (n = 8). **(E)** The percentage of infiltrating leukocyte subpopulations were measured (n = 8, ANOVA on Ranks, Student-Newman-Keuls). **(F-I)** TLR4-Expression in the paw was analyzed in leukocytes gated on CD45^+^ (**F**, x-axis: forward scatter, y-axis: CD45-PerCp-Cy5 fluorescence). TLR4-Expression in CD68^+^ M1 macrophages (**H**, x-axis: CD68-FITC, y-axis: TLR4-PE) and in CD163^+^ M2 macrophages (**I**, x-axis: CD163-FITC, y-axis: TLR4-PE) and compared to the unstained control (**G,** unstained control, x-axis: FITC fluorescence, y-axis: PE fluorescence). Representative examples are shown (n = 2).

In inflammatory infiltrate 4 d after CFA inflammation consisted of neutrophils and macrophages. Previously only a small number of infiltrating T-lymphocytes was observed by flow cytometry quantification (~5%) [22]. Since markers for proinflammatory M1 and antiinflammatory M2 macrophages are less defined in rats we used ED1 (CD68) and ED2 (CD163) as comparable markers [30]. CD163 is a scavenger receptor for the endocytosis and induced by antiinflammatory cytokines like IL-10. CD68 is a glycoprotein which binds to low density lipoprotein. In the inflammatory infiltrate in the paw after 4 d of inflammation the number of neutrophils (RP-1^+^CD45^+^) was significantly lower compared to CD68^+^CD45^+^ M1 proinflammatory or CD163^+^CD45^+^ M2 antiinflammatory cells (Figure 2A, B) while the percentages of CD68^+^CD45^+^ and CD163^+^CD45^+^ cells in the infiltrate were comparable. In conclusion, we did not observe a predominance of M2 vs. M1 macrophages. In the next step, we studied the expression of TLR4 in CFA inflammation (Figure 2C). The majority of CD45^+^ (>80%) cells in the paw expressed TLR4. Indeed, >75% of the CD68^+^CD45^+^ and >90% of the CD163^+^CD45^+^ cells were TLR4^+^.

### CD14^+^CD16^-^ anti-inflammatory monocytes contain more β-END released by TLR4 stimulation

**Figure 3:**
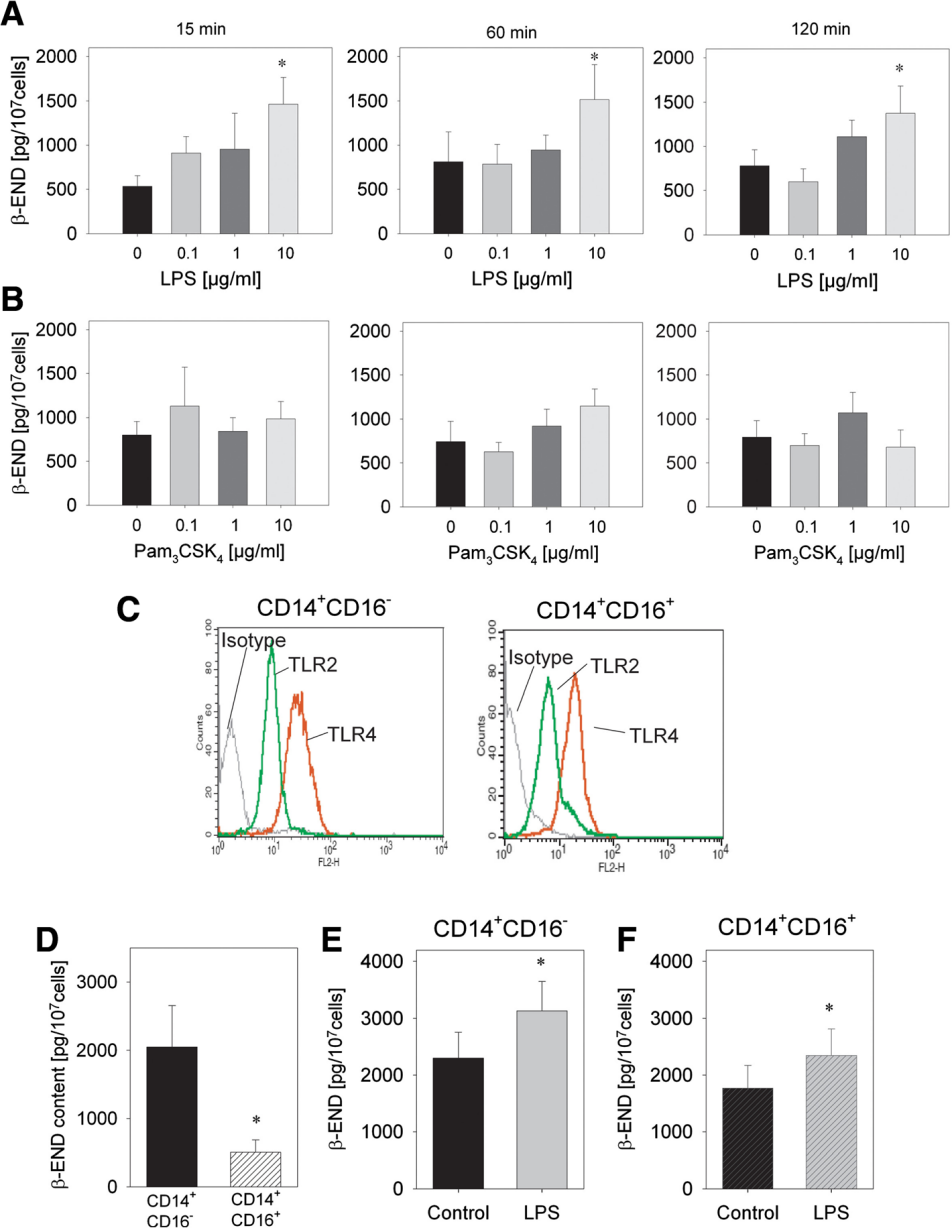
**Release of opioid peptides from monocytes in response to TLR4 but not TLR2 stimulation. (A, B)** Monocytes were isolated from human buffy coats and purified for CD14. Release of β-endorphin (β-END) was quantified after incubation for 15 or 120 min with different doses of LPS or Pam_3_CSK_4_ (n = 7–9, * p < 0.05, One way RM ANOVA, Student-Newman-Keuls). **(C)** Monocytes were separated into CD14^+^CD16^-^ and CD14^+^CD16^+^ monocytes. Expression of TLR2 (green line) and TLR4 (red line) was analyzed using flow cytometry in both populations. Isotype controls are shown (black line) (representative example, n = 3). **(D)** β-END content after lysis of unstimulated cells was measured by ELISA in these populations, CD14^+^CD16^-^ (black) and CD14^+^CD16^+^ (black striped) monocytes (n = 8, * p < 0.05 paired t-test) **(E,F)** CD14^+^CD16^-^ and CD14^+^CD16^+^ monocytes were stimulated for 15 min with 10 μg/ml LPS (red) compared to stimulation with solvent medium (black). β-END was analyzed in the supernatant (n = 8, * p < 0.05, paired t-test). All data are presented as MEAN ± SEM.

To evaluate release of opioid peptides and specifically β-END *in vitro* we used human monocytes due to availability in larger quantities. Incubation of purified CD14^+^ human monocytes with LPS, a potent agonist stimulating TLR4, induced a dose-dependent release of β-END after 15, 60 and 120 min (Figure 3A). A maximal significant release was observed with 10 μg/ml LPS resulting in a twofold increase. No release of β-END was seen after stimulation with Pam_3_CSK_4_ stimulating TLR2 independent of the time point (Figure 3B). In the next step we evaluated monocyte subpopulations: pro-inflammatory CD14^+^CD16^+^ and anti-inflammatory CD14^+^CD16^-^ purified by magnetic beads. Both CD14^+^CD16^+^ as well as CD14^+^CD16^-^ monocytes expressed TLR2 and TLR4 on the surface (Figure 3C). However, pro-inflammatory CD14^+^CD16^+^ contained significantly less β-END then anti-inflammatory CD14^+^CD16^-^ monocytes (Figure 3D). Both populations were able to release β-END after stimulation of LPS after 15 min (Figure 3E, F).

### β-END release by TLR4 stimulation is calcium-dependent

**Figure 4:**
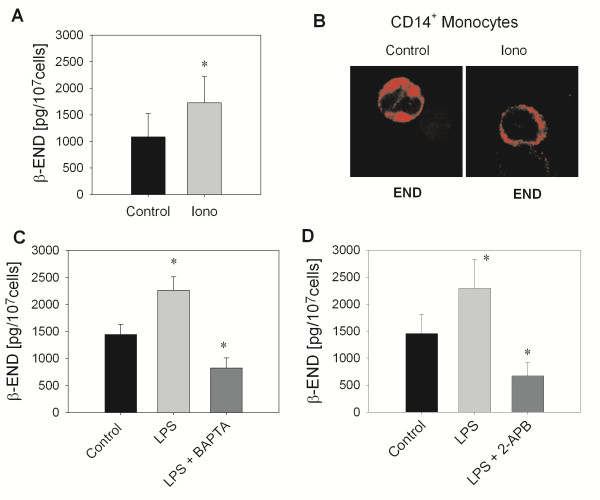
**Calcium requirement of TLR4-stimulated β-END release. (A)** Release of β-END in the supernatant was quantified after incubation of CD14^+^ monocytes for 15 min with 10 μM ionomycin (iono) or medium (control) (n = 3, * p < 0.05, paired t-test). **(B)** CD14^+^ monocytes were stimulated as described before and stained on cytospins using a ββ-END antibody (red) and confocal analysis (representative example, n = 3). **(C-E)** CD14^+^ monocytes were preincubated with BAPTA-AM (100 μM, n = 24–28, * p < 0.05, One Way RM ANOVA, Student-Newman-Keuls, **C)**, 2-APB (100 nM, n = 11 * p < 0.05, One Way RM ANOVA, Student-Newman-Keuls, **D)** for 10 min before the addition of 10 μg/ml LPS for 15 min. Secretion of β-END was quantified in the supernatant. All data are presented as MEAN ± SEM.

To study intracellular mechanism of opioid peptide release from monocytes we first analyzed β-END release after stimulation with the calcium ionophore ionomycin. Ionomycin elicited a significant release of β-END (Figure 4A) in the supernatant by 59.7% as well as a translocation of opioid peptides containing vesicles to the membrane as seen by confocal imaging (Figure 4B) indicating calcium-dependent release. To evaluate the mechanisms of LPS-dependent release, monocytes were preincubated with BAPTA-AM (Figure 4C), an intracellular Ca^2+^ chelator or 2-APB (Figure 4D), an IP_3_ receptor antagonist. Both treatments significantly inhibited LPS-induced β-END release. In addition, preincubation with BAPTA-AM as well as 2-APB significantly lowered release below baseline release. Therefore, release of opioid peptides by the TLR4 agonists LPS is dependent on the availability of intracellular calcium.

### *In vitro* differentiation of monocytes into macrophages increases β-END content in anti-inflammatory M2-macrophages

**Figure 5:**
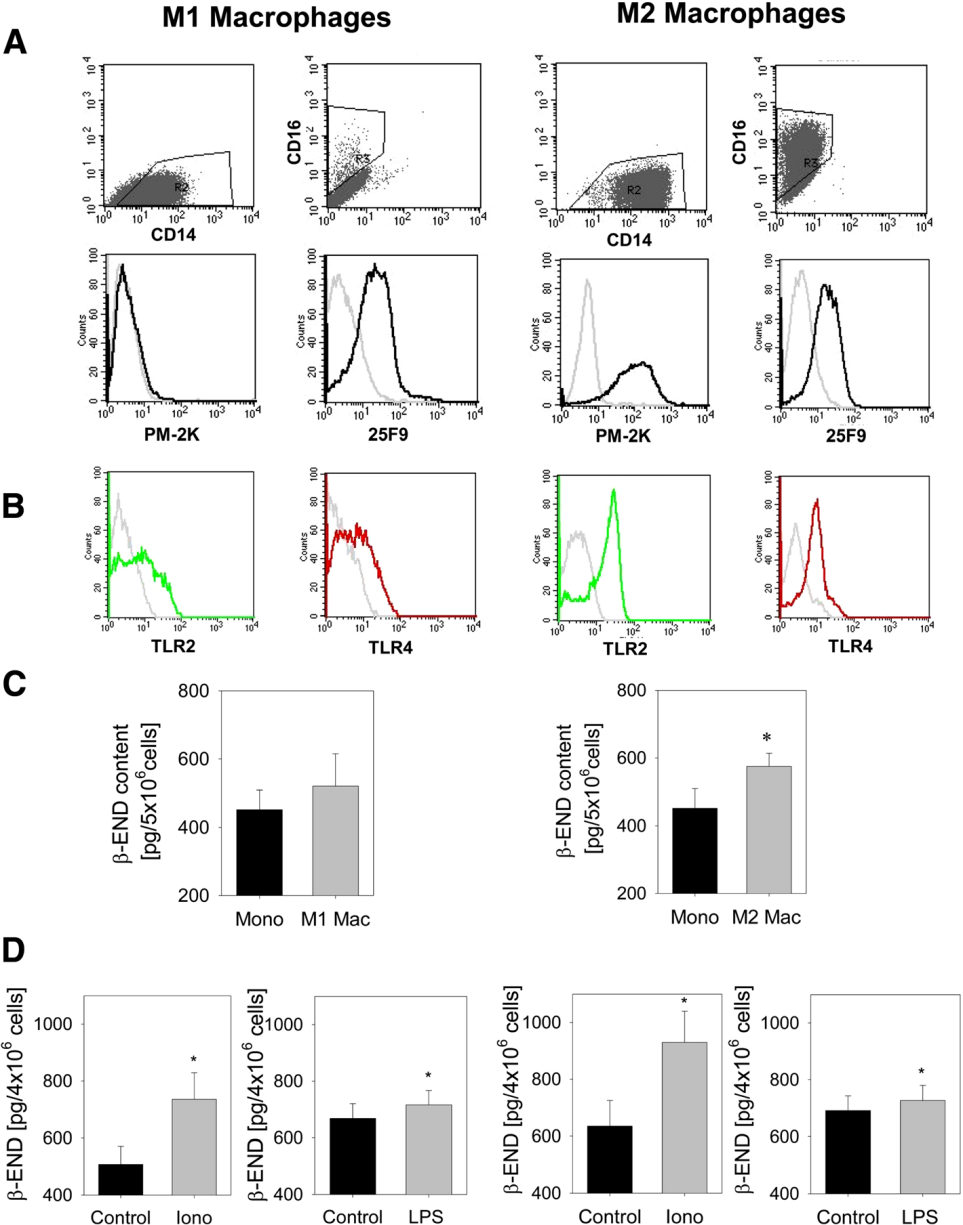
**Content and release of β-END in macrophage differentiation.** CD14^+^ monocytes were *in vitro* differentiated into M1 macrophages with GM-CSF (left column) and M2 macrophages with M-CSF and IL-10 (right column). Surface marker expression of CD16, CD14 as well as PM-2 K and 25 F9 as well as TLR2 and TLR4 were evaluated by flow cytometry. **(A)** Expression of CD16, CD14 as well as PM-2 K and 25 F9 on M1 and M2 macrophages as well as **(B)** TLR4 (red line) and TLR2 (green line). Representative examples were shown; isotype controls are depicted in grey lines (representative examples). **(C)** CD14^+^ monocytes (Mono) before differentiation and M1 and M2 macrophages (Mac) from the same individual after 6–7 d of culture were lysed and intracellular β-END content measured by ELISA (n = 14, * p < 0.05, paired t-test). **(D)** Release of β-END after incubation with 10 μM ionomycin (iono) or 10 μg/ml lipopolysaccharide (LPS) for 15 min from M1 and M2 macrophages was quantified in the supernatant (n = 21–24 for LPS and n = 7–8 for ionomycin, * p < 0.05, paired t-test). All data are presented as MEAN ± SEM.

When monocytes migrate into the tissue they differentiate into tissue macrophages. *In vitro* human monocytes differentiated into classical M1 macrophages by addition of GM-CSF and non-classical M2 macrophages by addition of M-CSF and IL-10 (Figure 5A). Human M1 macrophages expressed high levels of CD14 (86.5%) and low levels of CD16 (2.1%). In contrast human M2 macrophages were highly positive for both, CD14 (91.9%) and CD16 (84%). PM-2 K and 25 F9 are markers to characterize macrophages [25] PM-2 K is hardly expressed on M1 macrophages (mean: 1.8 ± 0.01%) but on most of M2 macrophages (mean: 77 ± 0.05%). 25 F9 is expressed on M1 macrophages as well as M2 macrophages (mean: 21.5 ± 0.06% vs. 66 ± 0.11%, respectively). TLR2 and TLR4 are expressed albeit in lower levels on M1 macrophages (TLR2: 21% and TLR4:18%) compared to M2 macrophages (TLR2: 60% and TLR4:14%) (Figure 5B).

Differentiation into human macrophages from the same donor resulted in an increase in opioid peptide content in M1 macrophages by 15% and significantly in M2 macrophages by 27.2% (Figure 5C). Ionomycin elicited secretion of β-END after 15 min (M1 macrophages by 45% and M2 macrophages 46.5%). In comparison, stimulation using LPS stimulated only a small release after 15 min (M1 macrophages by 5% and M2 macrophages 7.1%) (Figure 5D). Longer incubation times up to 120 min did not result in more release over time (data not shown). In summary, classical M1 macrophages produce less opioid peptides and expressed less TLR2 and TLR4 compared to non-classical M2 macrophages. Both subpopulations released β-endorphin in response to ionomycin and only minor amounts in response to LPS.

### TLR4-dependent antinociception of LPS in CFA inflammation and tonic analgesia

**Figure 6:**
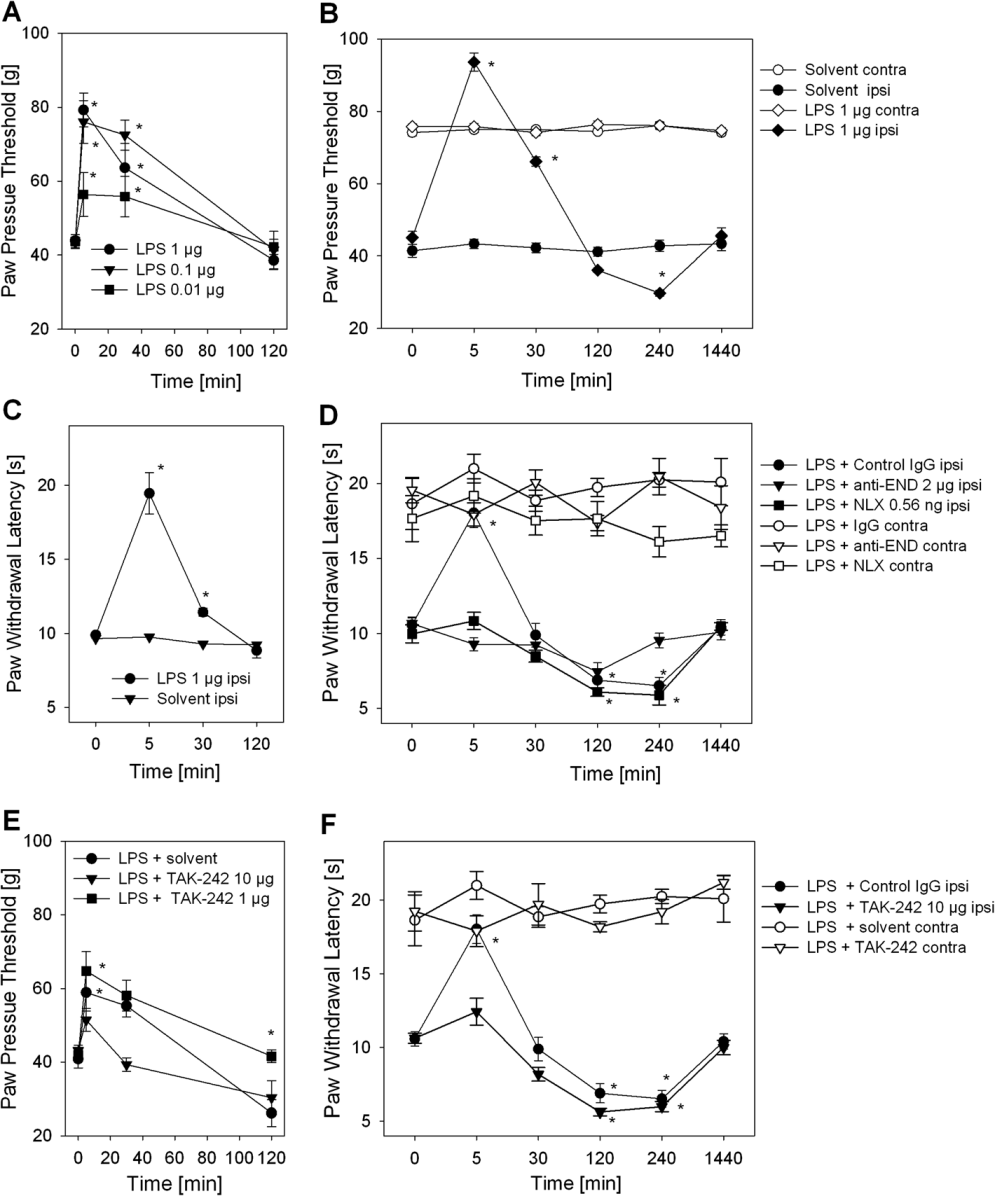
**Opioid-dependent LPS-induced antinociception in inflammatory pain.** All rats were injected with CFA i.pl. 4 d before further treatment. **(A)** Mechanical nociceptive thresholds were measured before and 5–120 min after injection of 100 μl LPS (0.01-1 μg) i.pl. (n = 6, * vs. 0 min, p < 0.05, Two way RM ANOVA, Student-Newman-Keuls). **(B, C)** Rats were treated with 1 μg LPS i.pl. and thermal nociceptive as well as mechanical thresholds were determined after indicated time points (n = 6, * vs. 0 min, * p < 0.05, Two way RM ANOVA, Student-Newman-Keuls). **(D)** Thermal nociceptive thresholds were measured after 5 min – 1 d in rats after 1 μg LPS i.pl. and as well as NLX (0.56 ng) or anti-β-endorphin antibody (anti-END, 2 μg) compared to control antibody (IgG2 μg, all black symbols). Contralateral paws from rats in each treatment group are shown for comparison (white symbols). **(E,F)** The effect of the small molecule TLR4 inhibitor, TAK-242 i.pl. (1, 10 μg/ml) on LPS-induced antinociception was evaluated in mechanical and thermal nociceptive thresholds. **(E)** Paw pressure thresholds in inflamed paws (ipsi, black symbols) were obtained after 5–120 min (n = 3–7). **(F)** Thermal nociceptive thresholds were measured in inflamed paws (ipsi, black symbols) rats after 1 μg LPS and 10 μg TAK-242 i.pl. at indicated time points compared to solvent (n = 6, * vs. 0 min, p < 0.05, Two way RM ANOVA, Student-Newman-Keuls). Contralateral (contra) paws are shown seen for comparison (white symbols). The data from the treatment group LPS + control IgG (black circles) is displayed for comparison from graph **6D**. All data are presented as MEAN ± SEM. always n = 6 except when indicated otherwise, * vs. 0 min, p < 0.05, Two way RM ANOVA, Student-Newman-Keuls).

In the last step, we evaluated the role of LPS and TLR4 *in vivo* in inflammatory pain. Rats with 4 d CFA hind paw inflammation were injected i.pl. with different doses of LPS. Mechanical nociceptive thresholds dose-dependently and significantly increased immediately after i.pl. LPS treatment indicating an antinociceptive property of LPS (Figure 6A). The optimal dose of LPS increased thermal nociceptive as well as mechanical thresholds (Figure 6B, C). Animals treated with LPS i.pl. did not show any signs of general illness and had no significant changes in mechanical or thermal nociceptive thresholds in the contralateral paw (Figure 6B, D). LPS-induced mechanical and thermal antinociception lasted up to 30 min and returned to baseline after 120 min. The antinociceptive effect of LPS was mediated by peripheral opioid receptors and opioid peptides (Figure 6D). Treatment with NLX i.pl. as well as anti-β-endorphin antibodies using peripheral selective doses [14] blocked LPS-induced thermal antinociception. No change in pain behavior was observed in contralateral paws. After the initial phase of antinociception LPS then significantly lowered thermal (Figure 6B) and mechanical (Figure 6D) nociceptive thresholds. LPS-induced hyperalgesia was unaffected by local anti-β-endorphin, but local naloxone reversed hyperalgesia.

**Figure 7:**
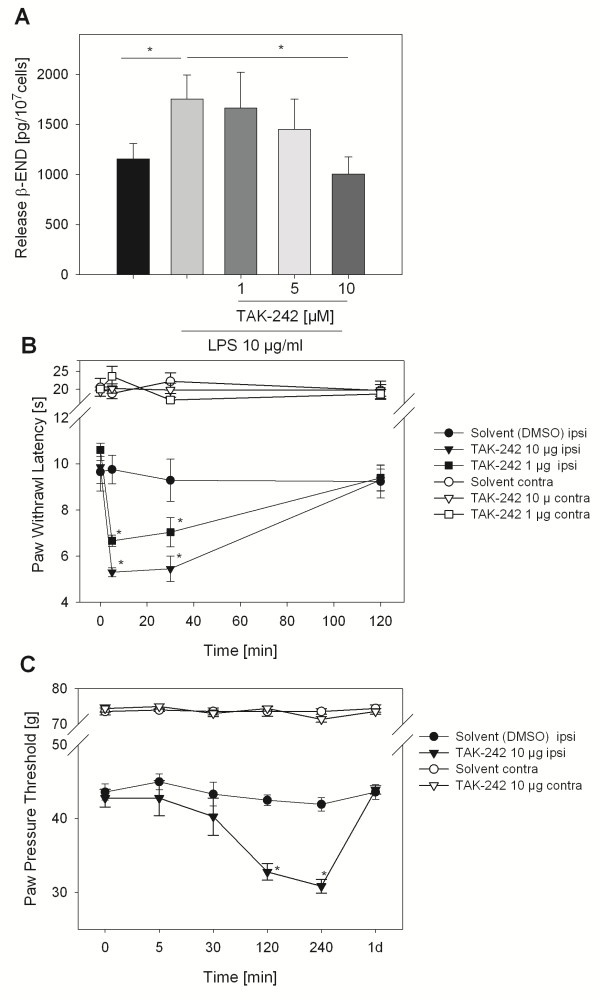
**Hyperalgesia by TLR4 blockade in inflammatory pain. (A)** Purified undifferentiated CD14^+^ human monocytes were incubated with 10 μg/ml LPS and a TLR4 inhibitor (TAK-242) in different doses. β-END release was quantified in the supernatant after 15 min (n = 6, * vs. LPS, *p <0.05, One Way RM ANOVA, Student-Newman-Keuls). **(B, C)** Rats with 4 d CFA inflammation were treated with TAK-242 i.pl. (1 μg, black triangle, and 10 μg, black square) and paw withdrawal latency **(B)** or paw pressure thresholds **(C)** were measured subsequently at indicated time points and compared to solvent (5% DMSO, black circle) in the inflamed paws (ipsilateral, ipsi). Paw pressure thresholds and paw withdrawal latencies from contralateral (contra, white symbols) paws are shown for comparison (n = 3–6, * vs. 0 min, * p < 0.05, Two way RM ANOVA, Student-Newman-Keuls). All data are presented as MEAN ± SEM.

To analyze the involvement of TLR4 in LPS-induced antinociception rats were concomitantly treated with a small molecule inhibitor of TLR4, TAK-242/resatorvid. Higher doses completely blocked LPS-induced mechanical (Figure 6E) and thermal (Figure 6F) antinociception and resulted in a further decrease of baseline hyperalgesia after 120 min. Nociceptive thresholds were unaffected in contralateral paws. *In vitro* TAK-242 was dose-dependently able to completely block LPS-induced β-END release in undifferentiated CD14^+^ human monocytes (Figure 7A). In the last step we wanted to determine of role of TLR4 in the generation of inflammatory hyperalgesia. The effect of TLR4 inhibition *in vivo* in rats with CFA inflammation on baseline thermal and mechanical nociceptive thresholds was analyzed. Treatment with TAK-242 resulted in a pronounced lowering of thermal nociceptive thresholds immediately after injection in rats with CFA inflammation, which lasted up to 30 min and returned to baseline after 120 min (Figure 7B). No change in the contralateral paws was observed in these animals. Mechanical nociceptive thresholds also dropped but with a different kinetics (Figure 7C). The maximal increase in hyperalgesia was seen after 240 min and returned to baseline after 1 day. Again contralateral paws were unaffected.

## Discussion

In the present study, peripheral opioid-dependent analgesia in the late phase is mediated via TLR4-stimulation of monocytes/macrophages releasing opioid peptides binding to peripheral opioid receptors. *In vitro*, monocytes and macrophages with an anti-inflammatory phenotype synthesize more opioid peptides. β-END is released from monocytes dependent on calcium through stimulation with the TLR4-agonist LPS. Intraplantar LPS injection *in vivo* immediately elicits opioid- and TLR4-dependent antinociception in CFA inflammation. In the later phase LPS further lowers nociceptive thresholds. Application of a TLR4-inhibitor in CFA inflammation further worsens baseline thermal and mechanical hyperalgesia.

### Peripheral opioid mediated analgesia

Different subpopulations of immune cells contribute to peripheral opioid-mediated antinociception. Neutrophils play an important role in the first phase because depletion of neutrophils in CFA inflammation or skin incision increases thermal hyperalgesia but not mechanical hyperalgesia [13-15]. Neutrophils are activated via chemokines or formyl peptides to secrete opioid peptides. Mobilization of neutrophils using GM-CSF treatment in CFA inflammation has no effect on mechanical hyperalgesia [31] and, on the contrary, even increases pain in neuropathic pain models [32].

Opioid-producing T cells are important soldiers in the battle against pain in different models including inflammatory pain [18,33] neuropathic pain [19] and visceral pain [20]. In CFA-induced arthritis antigen-specific activated, but not resting CD4^+^ T lymphocytes are responsible for the spontaneous relief of inflammation-induced pain following Ag challenge [18,33]. Analgesia was observed by transferring effector CD4^+^ T lymphocytes with Th1 or Th2 phenotype, suggesting that antinociceptive activity is a fundamental property of effector CD4^+^ T lymphocytes irrespective of their effector functions [18]. Similarly, in immunocompromised mice with chronic constriction neuropathy transfer of T-cells reverses neuropathic pain at later stages [19,34].

In later phases of CFA inflammation monocytes/macrophages are the major opioid-containing cells. Reduction (but not depletion) of monocytes/macrophages via clodoranate containing liposomes by 30% decreases opioid dependent swim stress-induced antinociception and has no effect on baseline hyperalgesia [23]. A higher selective depletion of monocytes/macrophages was not achieved. Therefore, we used the unselective immune depletion by CTX in this study. This treatment further worsened inflammatory mechanical hyperalgesia. Local application of μ-opioid receptor antagonists decreased thermal nociceptive thresholds. In accordance, mechanical nociceptive thresholds were lowered by intraplantar injection of naloxone or anti-β-END antibodies [29]. No change of nociceptive thresholds was observed in contralateral paws in this study or rats without inflammation in previous studies [28] arguing against a general proalgesic property of CTX.

Peripheral opioid-dependent analgesia is mostly short-term possibly because aminopeptidase N or neutral endopeptidases produced by leukocytes or peripheral nerves rapidly degrade opioid peptides [35]. Peripheral opioid-mediated analgesia lasts only shortly (around 10 min) in early inflammation when mostly neutrophils are triggered by chemokines or formyl peptides [13,14]. We now show that monocyte-based peripheral opioid-mediated analgesia via LPS lasts longer up to 30 min at 4 d after i.pl. CFA injection. Since both macrophages and neutrophils express activated aminopeptidase N or neutral endopeptidases we postulate that the longer duration of antinociception is due to a more sustained release. In accordance, we found that 15, 60 and 120 min stimulation of monocytes *in vitro* lead to significant opioid peptide release.

Our results are in contrast with a recent publication in mice with CFA inflammation [36]. In this model tissue injury produced μ-opioid receptor constitutive activity that repressed spinal nociceptive signaling for months. Therefore, NLX was able to prolong mechanical hyperalgesia but naltrexone methobromide, an opioid receptor antagonist that cannot cross the blood–brain-barrier, was not able to worsen mechanical hyperalgesia. In our model we used a standard peripheral selective dose of NLX. This excludes the possibility that central or spinal opioid receptors play a role in our model in rats. Also we observed differences in mechanical and thermal hyperalgesia. Therefore spinal and peripheral tonic opioid mechanisms exist but in part with a different kinetic and with different sensitivities (mechanical or thermal). Tonic opioid analgesia is also seen in other models like arthritic visceral pain [16] or visceral pain [17,37]. However, most groups have seen central nervous system effects of tonic opioids and not peripheral.

In summary, all leukocyte subpopulations contain and secrete opioid peptides in response to different stimuli. So far, there is no difference whether they belong to the innate or the specific immune system. Inflammatory pain and neuropathic pain in different models is worsened in immunosuppressive states.

### Monocyte and macrophage subtypes

In our study we did not find a difference of TLR expression in CD14^+^CD16^+^ compared to CD14^+^CD16^-^ monocytes in healthy donors. In contrast, patients with acute myocardial inflammation, TLR4 were more expressed on circulating CD14^+^CD16^+^ monocytes than on CD14^+^CD16^-^ monocytes [24]. Differentiation into M1 or M2 macrophages, however, decreased TLR2 and TLR4 expression. Functional response to TLR4 stimulation, specifically opioid peptide release, revealed no difference between pro-and anti-inflammatory monocytes, although anti-inflammatory monocytes seemed to contain more opioid peptides. Elevated blood levels of inflammatory monocytes (CD14^+^CD16^+^) are present in patients with complex regional pain syndrome [38]. Lower levels of opioid peptide content could support increased pain in these patients. *In vitro* differentiated M1 and M2 macrophage were hardly able to release β-END in response to TLR4 stimulation. This might be partially related to the decreased levels of TLR4 expression under *in vitro* culture conditions, higher amounts of opioid peptides released in response to “priming” by cytochalasin or the necessity of co-stimulatory molecule activation [39]. In the inflamed paw both M1 (CD68^+^) and M2 (CD163^+^) macrophages were found with no obvious predominance of one type. However, characterization of polarized macrophages *in vivo* in the tissue has remained difficult and data have to be interpreted with caution, because a battery of cell markers might be necessary to distinguish between these populations *in vivo*[30]. Nevertheless, both macrophage subpopulations were highly positive for TLR4. *In vitro* differentiated human M1 macrophages were less positive for TLR4. In summary, TLR4 is expressed on all monocyte and macrophage subpopulations to a various extent. TLR4 stimulation releases opioid peptides, but *in vitro* differentiated macrophages seem to be much less responsive.

TLR4 stimulates two intracellular cascades. The major one involves MyD88, IL-1R associated kinase (IRAK), tumor necrosis factor associated factor 6 (TRAF6) and NFĸB or mitogen-activated proteins kinas (MAPK). Activation of MAPK pathways causes release of inflammatory mediators like chemokines [3]. Calcium-dependent processes support this. TLR4, -9, and −3 ligands markedly induce intracellular calcium fluxes and activate CaMKII-alpha in macrophages [40]. Calcium has been shown to be essential for opioid peptide release via formyl peptide receptor of CXCR2 in neutrophils [13,14]. Similar mechanisms seem to be important in monocytes. However, there are differences in the amount of opioid peptides released and the duration of release. Monocytes/macrophages release lower amounts *in vitro* compared to an up to fourfold increase in opioid peptide release from neutrophils after formyl peptide stimulation [14]. In neutrophils, opioid peptide secretion is rapid (after 7 min) with no further increase, while in monocytes releases is detectable up to 120 min. Similarly in *in vivo* studies i.pl. injection of formyl peptides in 2 h CFA inflammation induces an antinociceptive effect for 10 min [14] while in this study i.pl. LPS increases mechanical nociceptive thresholds for 30 min in 96 h CFA inflammation.

### Role of TLRs in pain

Most of the studies in TLR and pain have concentrated on the nociceptive processing in the spinal cord. Following tissue insult and nerve injury, TLRs (such as TLR2, TLR3, and TLR4) induce the activation of microglia and astrocytes and the production of the proinflammatory cytokines in the spinal cord, leading to the development and maintenance of inflammatory pain (e.g. serum-transferred arthritis) and neuropathic pain [3,41,42]. In neuropathic pain, both the genetically altered mice and the rats treated with TLR4 antisense oligodeoxynucleotide have a significantly attenuated behavioral hypersensitivity and decreased expression of spinal microglial markers and proinflammatory cytokines compared to their respective control groups [8]. This is also observed in rats with bone cancer pain were treated intrathecally with TLR4 siRNA [43]. Stimulation of murine dorsal root ganglion neurons with TLR ligands (TLR3, 7, and 9) induced expression and production of proinflammatory chemokines and cytokines, upregulation of transient receptor potential vanilloid type 1 (TRPV1), and enhanced calcium flux by TRPV1-expressing dorsal root ganglion neurons [4]. TLR4 is also implicated in spinal opioid-induced hyperalgesia [44]. Morphine binds to an accessory protein of TLR4 thereby inducing TLR4 oligomerization and triggering spinal inflammation. In summary, TLR4 on microglia significantly modulates several types of pain and morphine treatment finally leading to hyperalgesia.

Much less is known about the role of TLR4 in the periphery. In our studies i.pl. injection of LPS elicits a transient dose-dependent mechanical and thermal antinociceptive effect blocked by a TLR4 antagonist. This is followed in the later phase by a drop in thermal and mechanical nociceptive thresholds. This second phase of hyperalgesia is in line with other studies demonstrating that LPS elicits hyperalgesia in rats in naïve animals [45]. Similarly, in healthy volunteers, activation of TLR4 by endotoxin caused significant increase in capsaicin-induced allodynia and hyperalgesia following intradermal capsaicin [46]. Intrathecal injection of LPS in the lumbar spinal cord produces mechanical hyperalgesia in rat hind-paws dependent on purigergic receptors [47]. Therefore pro-versus analgesic properties of LPS seem to be dependent on the context and the time, the anatomical location and the model used.

TAK-242 binds selectively to TLR4 and subsequently disrupts the interaction of TLR4 with adaptor molecules, thereby inhibiting TLR4 signal transduction and its downstream signaling event [48]. In contrast to the above-described studies highlighting the antinociceptive properties of TLR-antagonists we here demonstrate that TLR4 blockade immediately worsens thermal hyperalgesia in inflammatory pain. Indeed, we postulate that the proalgesic action of TAK-242 is due to peripheral inhibition of tonic opioid peptide release. Endogenous TLR ligands (danger associated adapter proteins, DAMPS) are for example heat shock proteins 22, 60, 70 and 72, high-mobility group box protein B1 (released after cell necrosis), oxidized low sensitive of lipoprotein or fibronection in tissue repair. All of them are found in inflamed tissue [3] and could contribute to peripheral opioid-mediated analgesia by macrophages in inflamed tissue. Furthermore TLR4 blockade worsens mechanical hyperalgesia with a delay. This could be better explained by effects on central sensitization. Therefore, we propose that different peripheral and spinal mechanisms are to be responsible for the increased hyperalgesia with TLR4 blockade.

## Conclusion

In late CFA inflammation monocytes/macrophages are responsible for peripheral opioid-mediated analgesia. Endogenous TLR4 agonists could stimulate opioid peptide release from monocytes and contribute to inflammatory pain control. Antiinflammatory monocytes/macrophages seem to be more important in this respect. Taken together we provide further evidence that both immunosuppressive treatment – regularly used in the treatment of e.g. in autoimmune disease – or anti-TLR4 treatment impair endogenous pain control mechanisms.

## Methods

### Animals

Animal protocols were approved by the animal care committees (Landesamt für Arbeitsschutz, Gesundheitsschutz und Technische Sicherheit, Senate of Berlin, und Regierung von Unterfranken, Germany), and are in accordance with the International Association for the Study of Pain [49]. Male Wistar rats weighing 180–220 g were treated as described below under brief isoflurane anesthesia. Animals were sacrificed using intracardial injection of a solution of T61 (embutramide, mebezonium and tetracaine) under isoflurane anesthesia according to national guidelines.

### Nociceptive thresholds and paw volume

Mechanical nociceptive thresholds were determined using the paw pressure algesiometer (modified Randall-Selitto test; Ugo Basile, Comerio, Italy) as described before [50]. A blunt piston onto the dorsal surface of the hind paw applied the pressure. The pressure required to elicit paw withdrawal was defined as the paw pressure threshold. The average of three measurements was calculated. Treatments were randomized and blinded. A decrease in the paw pressure threshold was interpreted as pain (hyperalgesia) whereas a rise in the paw pressure threshold was interpreted as analgesia (antinociception).

Thermal nociceptive thresholds were measured by the Hargreaves test as previously described [50]. The latency (time; s) required to elicit paw withdrawal was measured with an electronic timer (IITC Inc/Life Science, Woodland Hills, CA, USA) after application of radiant heat to the plantar surface of a hind paw from underneath the glass floor with a high-intensity light bulb. The stimulus intensity was adjusted to 20 s for the paw withdrawal latency in non-inflamed paws, and a cutoff of 30 s was set to avoid tissue damage. The average of two measurements taken with 20 s intervals was calculated. A decrease in paw withdrawal latency was interpreted as pain (hyperalgesia) whereas a rise in paw withdrawal latency was interpreted as analgesia (antinociception)

Paw volume was measured by submerging the hind paw till the tibiotarsal joint inside the water-filled Perspex cell of a plethysmometer at the same time points (37140, Ugo Basile, Comerio, Italy) [51].

### Treatments

Rats were intraplantarly (i.pl.) injected into the right hind paws with 150 μl CFA (Calbiochem, Darmstadt, Germany) for 4 d. Separate groups of rats were treated with i.pl. injection of LPS, naloxone (NLX) (both Sigma-Aldrich, St. Louis, MO, USA) or TAK-242, resatorvid, a small molecule TLR4 inhibitor (MedChem Express, HY-11109, Princeton, NJ, USA). Doses were chosen according to pilot experiments or previous experiments [50,52].

For immunodepletion rats were treated with cyclophosphamide (CTX) (Baxter, Unterschleissheim, Germany) according to an established protocol [27], because selective monocyte/macrophages depletion using clodronate has previously been unsuccessful [23]. Animals were injected i.p. 3 d and 1 d before the experiment with 100 mg/kg and 50 mg/kg of cyclophosphamide as well as on day two after CFA i.pl. This treatment results in over 90% reduction in circulating leukocytes [26]. No change in basal nociceptive thresholds after this treatment has been observed before [28].

### Monocyte isolation and macrophage differentiation

Human monocytes were used because of lack of sufficient quantity and purity of rat blood monocytes to be used for in vitro stimulation [53,54]. Monocytes were isolated from leukocyte apheresis filters of healthy donors by magnetic cell separation using anti-CD14-microbeads (Miltenyi Biotec, Bergisch Gladbach, Germany) or a combined purification using anti-CD16-microbeads immediately after harvest. Due to anonymous collection from left over apheresis filters, no consent from donors was necessary in line with the ethical committee of the institution. Human monocytes were cultured in RPMI (Invitrogen, Darmstadt, Germany) supplemented with 10% fetal calf serum (PAA Laboratories, Cölbe, Germany), 100 μg/ml streptomycin and 100 units/ml penicillin (both Biochrom AG, Berlin, Germany).

CD14^+^ human monocytes were cultured in a density of 2 × ∁10^5^ cells/cm^2^ in CellBIND surface cell culture flasks for 6–7 days with 50 ng/ml GM-CSF to generate GM- or M1-macrophages and 50 ng/ml M-CSF and 25 ng/ml IL-10 to generate M- or M2-macrophges according to the literature [55].

### Opioid peptide content and release

1×10^7^ CD14^+^ monocytes or 0.5×10^7^ M1 or M2 macrophages were stimulated with LPS (1–10 μg/ml, Sigma-Aldrich), Pam3CSK4 (1–10 μg/ml, InvivoGen, San Diego, USA) or ionomycin (10 μM) after preincubation with cytochalasin B (dissolved in dimethyl sulfoxide, final 5 μg/ml) for 5 min in Hank’s balanced salt solution containing the proteinase inhibitors bestatin (40 μg/ml), aprotinin (10 μg/ml) and thiorphan (dissolved in dimethyl sulfoxide, final 25 μg/ml, all Sigma-Aldrich) [14]. Doses of LPS, Pam3CSK4 and ionomycin were based on pilot experiments and the literature [14]. In some experiments, cells were concomitantly incubated with inhibitors: 1, 2-Bis(2-aminophenoxy)ethane-N,N,N’,N’-tetraacetic acid tetrakis acetoxymethyl ester (BAPTA-AM, dissolved in dimethyl sulfoxide, Calbiochem, Merck4Biosciences, Darmstadt, Germany) is a cell-permeant Ca^2+^ chelator used to control the level of intracellular Ca^2+^. 2-Aminoethoxydiphenyl borate (2-APB, dissolved in dimethyl sulfoxide, Calbiochem) inhibits IP_3_ receptors. In previous experiments the solvent dimethyl sulfoxide (maximal final concentration 1%) did not induce significant release [14]. Supernatants were obtained after 15–120 min stimulation in accordance with the rapid effect of these mediators in pain behavior tests.

For the measurement of opioid peptide content 1×10^6^ M1 and M2 macrophages were resuspended in ELISA buffer followed by five freeze (10–20 s) - thaw (90 s) cycles using liquid nitrogen and a 37°C water bath as well as subsequent vortexing as described before [29]. After centrifugation to remove unsolvable material the supernatants containing opioid peptides were obtained. Supernatants were stored at −20°C until further analysis by radioimmunoassay using commercially available kits for human β-END (Phoenix Pharmaceuticals, Burlingame, USA).

### Flow cytometry

Cellular staining of monocytes or macrophages was performed as previously described [50]. Single cell suspensions were incubated with the following primary and secondary antibodies (all antibodies by BD Biosciences, Heidelberg, Germany unless stated otherwise): Macrophages were preincubated with Fc block (mouse anti-rat CD32) for 5 min at 4°C, then the first antibody anti-human, PM-2 K (10 μg/ml, AbD Serotec, Oxford, Great Britain), anti-human 25 F9 Biotin (10 μg/ml, ebioscience, Frankfurt, Germany) or isotype control antibodies were added for 30 min at room temperature. After washing cells were incubated with the second antibody for 30 min (anti-mouse IgG_1_ FITC or Streptavidin PE-Cy5). TLRs were labeled on human monocytes or macrophages using PE conjugated anti-human-TLR2, anti-human-TLR4 (ebioscience) or isotype control antibodies according to manufacturer’s instructions. CD14 and CD16 were stained with FITC-conjugated anti-CD14 and PE-conjugated anti-CD16 antibodies for 15 min (both from Miltenyi Biotec, Bergisch Gladbach, Germany).

For tissue analysis, subcutaneous paw tissue was obtained and digested as described before [26,56]. Cell suspension were stained with a mouse monoclonal anti-rat-CD45-PerCP-Cy5, anti-rat-RP-1-PE (both BD bioscience), anti-rat-CD68-FITC and anti-rat-CD163-FITC (both AbD Serotec) and mouse monoclonal anti-rat-TLR4-PE (Thermo Scientific Pierce Product, Bonn, Germany). For RP-1 and CD68 double staining cells were first fixed in paraformaldehyde and permeabilized in saponin as previously described [26,56].

The FACS Scan acquired 10.000-20.000 FACS events. Data analysis was performed by CellQuest software (all BD Biosciences).

### Immunofluorescence

CD14^+^ human monocytes were preincubated for 5 min with cytochalasin B followed by stimulation with 10 mM ionomycin for 15 min. Cells were harvested and centrifugated. Cell pellets of monocytes were reconstituted in 5 ml PBS, and 50,000 monocytes in suspension were then centrifuged by a Shandon Cytospin 3 (Thermo Shandon, Pittsburgh, PA) at 20 g for 3 min on glass slides. Monocytes were fixed for 30 min and confocal analysis was carried out as previously described [14,57]. Briefly, monocytes cytospins were incubated with rabbit polyclonal antibodies against β-END (1:1000, Peninsula Laboratories, Belmont, CA, USA) and subsequently with a Texas red-conjugated goat anti-rabbit antibody. Thereafter, cytospins were washed with PBS and mounted in vectashield. Images were acquired on a Zeiss LSM510META confocal laser scanning system (Zeiss AIM; Jena, Germany) using a 63×/1.4 Plan-Apochromat or 40×/1.3 Plan-Neofluar oil immersion objective in a series of optical sections of about 1 μm thickness. Each experiment was repeated three times. To demonstrate specificity of staining, the following controls were included as mentioned in detail elsewhere: (1) preabsorption of diluted antibody against β-END with purified β-END (Peninsula laboratories-Bachem) and (2) omission of either the primary or the secondary antibodies.

### Statistical analysis

Data are presented as raw values (mean ± SEM). Data were tested for normality and for equal variance. Normally distributed data were analyzed by t-test or paired t-test for samples before and after treatment. If aliquots of one sample were exposed to different conditions or repeated measurements from one animal were taken, One or Two Way repeated measurement (RM) ANOVA was used. The post hoc comparisons were performed by Student-Newman-Keuls. In case of not normally distributed data the test was performed on ranks. Differences were considered significant if p < 0.05.

## Abbreviations

2-APB: 2-Aminoethoxydiphenyl borate; BAPTA: Bis(2-aminophenoxy)ethane-N,N,N’,N’-tetraacetic acid tetrakis acetoxymethyl ester; CFA: Complete Freund’s adjuvans; CTX: Cyclophosphamide; β-END: β-endorphin; DMSO: Dimethyl sulfoxide; LPS: Lipopolysaccharide; Met-ENK: Met-enkephalin; NLX: Naloxone; TLR: Toll like receptors; TRPV1: Transient receptor potential vanilloid type 1.

## Competing interests

The authors declare that they have no competing interests.

## Authors’ contributions

RSS and DH designed, carried out and analyzed the pain behaviour experiments and participated in the manuscript draft. HS and LM performed and analyzed the *in vitro* studies with monocytes/macrophages. YW performed some FACS experiments. SAM carried out the immunostaining. NR participated in the design of the study. HR and AB designed, coordinated and wrote the manuscript. All authors read and approved the final manuscript.
